# The Influence of Weather and Lemmings on Spatiotemporal Variation in the Abundance of Multiple Avian Guilds in the Arctic

**DOI:** 10.1371/journal.pone.0101495

**Published:** 2014-07-01

**Authors:** Barry G. Robinson, Alastair Franke, Andrew E. Derocher

**Affiliations:** 1 Department of Biological Sciences, University of Alberta, Edmonton, Alberta, Canada; 2 Canadian Circumpolar Institute, University of Alberta, Edmonton, Alberta, Canada; University of Lleida, Spain

## Abstract

Climate change is occurring more rapidly in the Arctic than other places in the world, which is likely to alter the distribution and abundance of migratory birds breeding there. A warming climate can provide benefits to birds by decreasing spring snow cover, but increases in the frequency of summer rainstorms, another product of climate change, may reduce foraging opportunities for insectivorous birds. Cyclic lemming populations in the Arctic also influence bird abundance because Arctic foxes begin consuming bird eggs when lemmings decline. The complex interaction between summer temperature, precipitation, and the lemming cycle hinder our ability to predict how Arctic-breeding birds will respond to climate change. The main objective of this study was to investigate the relationship between annual variation in weather, spring snow cover, lemming abundance and spatiotemporal variation in the abundance of multiple avian guilds in a tundra ecosystem in central Nunavut, Canada: songbirds, shorebirds, gulls, loons, and geese. We spatially stratified our study area based on vegetation productivity, terrain ruggedness, and freshwater abundance, and conducted distance sampling to estimate strata-specific densities of each guild during the summers of 2010–2012. We also monitored temperature, rainfall, spring snow cover, and lemming abundance each year. Spatial variation in bird abundance matched what was expected based on previous ecological knowledge, but weather and lemming abundance also significantly influenced the abundance of some guilds. In particular, songbirds were less abundant during the cool, wet summer with moderate snow cover, and shorebirds and gulls declined with lemming abundance. The abundance of geese did not vary over time, possibly because benefits created by moderate spring snow cover were offset by increased fox predation when lemmings were scarce. Our study provides an example of a simple way to monitor the correlation between weather, spring snow cover, lemming abundance, and spatiotemporal variations in Arctic-breeding birds.

## Introduction

Spatial and temporal variation in the abundance of organisms is of central importance to the study of ecology [1], particularly in the face of environmental change [2]. Climate change, one of the most significant environmental perturbation occurring today, can have a strong influence on the distribution and abundance of organisms by altering the trophic interactions within a community [3]–[5]. In Arctic ecosystems, where the time available for breeding is short and the food chain is relatively simple, altered trophic interactions may be more critical than at southern latitudes [6], [7]. In addition, some of the most severe changes in climate are occurring in Arctic ecosystems: temperatures are rising at almost twice the rate of the rest of the planet and summer rainfall has increased significantly over the last century [8], [9]. Particular attention should, therefore, be paid to the influence of climate on trophic interactions within Arctic communities.

In experimental and natural systems, the negative effects of a warming climate are exacerbated in species at higher trophic levels [10], [11] because these species adjust their phenology with climate change more slowly than species at lower trophic levels [12]–[15]. Asynchrony in phenological change can create a mismatch between the needs of a predator and the availability of their prey [16]. Herbivorous and insectivorous Arctic-breeding birds feeding at low trophic levels may be most susceptible to phenological mismatch because their food responds quickly to changing weather patterns. Climate warming was associated with an advance in the annual summer pulse in arthropod abundance, making it asynchronous with the hatching of insectivorous shorebird chicks, which experienced reduced growth rates [17], [18]. A mismatch between the timing of vegetation green-up and the hatching of herbivorous snow geese (*Chen caerulescens*) resulted in lower gosling body condition and first-year survival [19], [20]. Gauthier et al. [21] found a similar mismatch between the phenology of snow geese and tundra vegetation, but they did not find any evidence for reduced productivity or abundance of geese.

Climate change in the Arctic can alter trophic interactions between birds and their prey even if their respective phenologies are unaffected. Declines in the persistence of summer sea ice reduced Arctic cod (*Boreogadus saida*) abundance causing several marine bird species to switch to alternative prey [22]–[24]. In addition to warming temperatures, summer rain storms in the Arctic are predicted to become more frequent and severe under most climate models [8], which may reduce foraging opportunities for insectivorous shorebirds [25], [26].

Warming temperatures in the Arctic can also lead to benefits for birds, potentially countering negative effects caused by altered trophic interactions. Up to half of the energy metabolized by shorebird chicks is used for feeding and thermoregulation [27], so Arctic-breeding shorebirds could benefit from warming temperatures associated with climate change. McKinnon et al. [28] found that even when forage availability was below average, dunlin (*Calidris alpina*) chicks in the sub-Arctic were able to maintain above average growth rates with increasing summer temperatures. Snow geese spent less time brooding their young as temperatures increased, allowing more time and energy to be allocated to foraging [29]. Warmer Arctic temperatures are also reducing spring snow cover [30], increasing nest density, nest success and overall productivity for a variety of goose species in different circumpolar regions [20], [31], [32]. In geese and other Arctic-breeding birds, earlier spring snow melt results in earlier nest initiation [33]–[35], which can increase clutch size, and nestling growth and survival [36], [37].

Trophic interactions independent of weather, such as predation, can also influence the abundance of Arctic-breeding birds. Arctic foxes (*Vulpes lagopus*) prey primarily on collared lemming (*Lemmus trimucronatus*) and brown lemming (*Dicrostonyx groenlandicus*) when lemmings are abundant, but switch to their alternate prey of bird eggs when lemming abundance declines [38]. Lemming populations cycle throughout the Arctic peaking every 3–5 years, so Arctic fox predation rates on bird eggs also fluctuate [39]. In the Canadian and Russian Arctic, population size, clutch size, egg survival and nest success of multiple species of geese and shorebirds all correlated positively with lemming abundance, declining when lemming populations crashed and Arctic foxes began consuming bird eggs [40]–[44]. Predictions made under various climate change scenarios indicate the amplitude and frequency of peaks in the lemming cycle are likely to decrease [45], which may increase predation pressure on Arctic-breeding birds. The complex interactions between summer temperature, rain, spring snow cover, phenology, thermoregulation, and forage availability, coupled with fluctuations in predation pressure, hinder our ability to accurately predict how Arctic-breeding birds will respond to climate change.

The main objective of this study was to investigate the relationship between annual variation in weather and lemming abundance, and spatiotemporal variation in the abundance of multiple avian guilds in an Arctic tundra ecosystem in central Nunavut, Canada: songbirds (Passeriformes), shorebirds (Scolopacidae and Charadriidae), gulls (Laridae, Sternidae and Stercorariidae), loons (Gaviiformes), and geese (Anatidae). Species within each guild occupy similar ecological niches in terms of diet and foraging habitat (see Table 1 for references), so we expected similar responses to landscape and weather variables within each guild. We hypothesized that spatial variation in the abundance of all guilds was correlated with some combination of vegetative productivity, topography, and the abundance of freshwater (Table 1). We also hypothesized that abundance of all guilds was positively correlated with mean summer temperature and negatively correlated with summer rainfall. Although a general warming climate may result in phenological mismatch across trophic levels over a longer temporal scale (e.g., decades), we predicted the short-term influence of warm weather and low rainfall would result in higher bird abundance, potentially due to increased feeding opportunities, reduced costs of thermoregulation, and decreased spring snow cover. Finally, we hypothesized that the abundance of all guilds was positively correlated with lemming abundance. Understanding the relationship between weather, the lemming cycle, and avian abundance will provide additional insight into the sensitivity of Arctic-breeding birds to climate change.

**Table 1:** *A priori* hypotheses predicting the relationship between the abundance of different avian guilds and three landscape metrics.

Guild	Hypotheses		
	Vegetation	Topography	Standing freshwater
Songbirds [74], [76], [91]	abundance positively correlated with productivity	more abundant in flat vs. rugged habitats	neutral
Shorebirds [92]–[95]	abundance positively correlated with productivity	more abundant in flat vs. rugged habitats	abundance positively correlated with amount of standing water
Gulls [72], [80], [96], [97]	neutral	neutral	abundance positively correlated with amount of standing water
Geese [98]–[100]	abundance positively correlated with productivity	neutral	abundance positively correlated with amount of standing water
Loons [70], [101], [102]	neutral	neutral	abundance positively correlated with amount of standing water

## Methods

### Ethics statement

All of our field methods were reviewed and approved by the University of Alberta's Animal Care and Use Committee (protocol number 738), the Government of Nunavut's Department of Environment (Wildlife Research Permit numbers 2010–009, 2011–038 and 2012–042), and the Igloolik Hunters and Trappers Association.

### Study area

**Figure 1:**
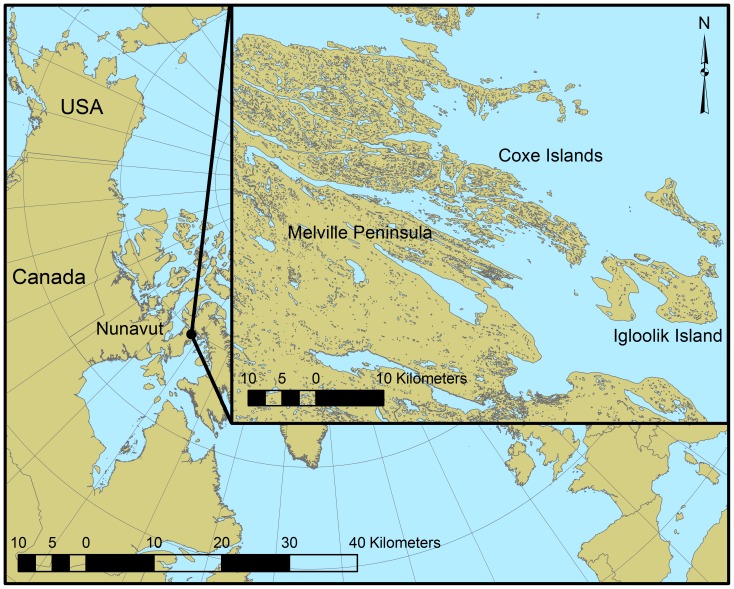
Study area location near Igloolik, Nunavut, Canada. Transects surveyed for birds were located throughout the Coxe Islands, Igloolik Island, and the northern tip of the Melville Peninsula.

**Figure 2:**
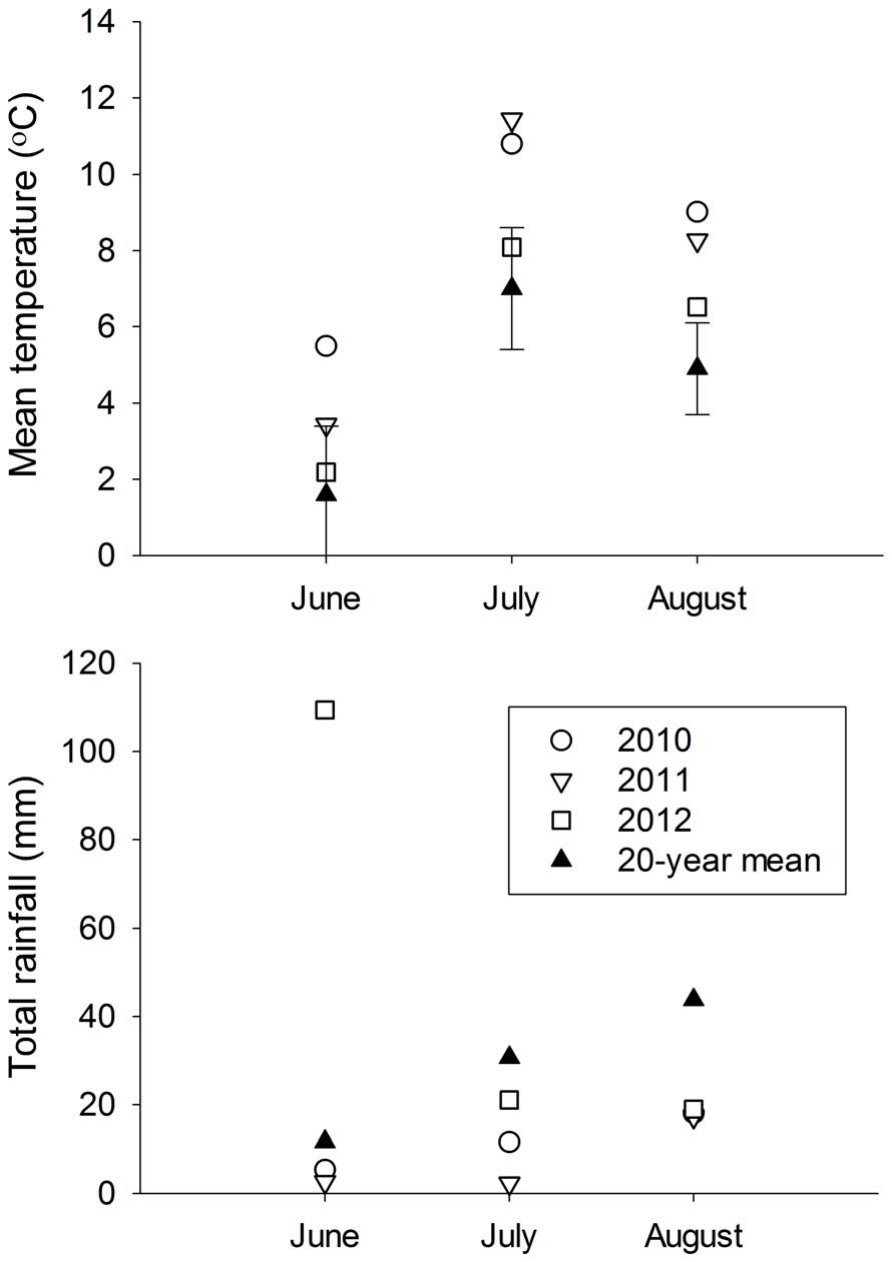
Mean monthly temperature and total monthly rainfall in the Igloolik study area. Data from 2010–2012 were collected during this study using a remote weather station located on the Coxe Islands. The 20-year mean is based on data from 1980–2000 collected by Environment Canada at the Igloolik airport (http://www.weather.gc.ca). Error bars represent standard deviations of monthly mean temperatures over the 20-year time period. Environment Canada does not report error for the 20-year mean of rainfall.

This study was conducted in the Northern Arctic Ecozone [46] near the community of Igloolik, Nunavut, Canada, among the Coxe Islands, Igloolik Island and the northern tip of the Melville Peninsula (Figure 1: 69.5345°N, 82.5070°W). The region has short cool summers with monthly mean temperatures fluctuating from 1.6 to 7.0°C (Figure 2). Spring thaw begins in early June and the majority of snow cover on land is melted by early July. Sea ice persistence varies annually, but most ice is generally gone by mid-late July. Summer rainfall is generally low averaging 86 mm from June to August (Figure 2). The study area covered 2030 km^2^ of rugged coastline, rolling tundra, and ocean (57% of area). Cliffs are numerous throughout the area, generally occurring along the shore of the ocean or large inland lakes. Cliffs provide suitable nesting habitat for raptors such as peregrine falcons (*Falco peregrinus*), gyrfalcons (*F. rusticolus*), and rough-legged hawks (*Buteo lagopus*) as well as common ravens (*Corvus corax*), glaucous gulls (*Larus hyperboreus*), Thayer's gulls (*L. thayeri*), Canada geese (*Branta canadensis*), and common eiders (*Somateria mollissima*). Black guillemot (*Cepphus grylle*) colonies occur on rocky shorelines and small rocky islands. Inland rolling tundra is vegetated with lichens, mosses, graminoids (e.g., *Luzula* spp., *Carex* spp., *Alopecurus magellanicus*, and *Poa* spp.), herbs (e.g., *Saxifraga* spp., *Bistorta vivipara*, and *Pedicularis* spp.) and low shrubs (*Salix* spp., *Dryas* spp., and *Cassiope tetragona*). Small lakes and wetland areas are numerous throughout the tundra providing habitat for a diverse community of songbirds and shorebirds such as Lapland longspurs (*Calcarius lapponicus*), snow buntings (*Plectrophenax nivalis*), American golden-plovers (*Pluvialis dominica*), semipalmated plovers (*Charadrius semipalmatus*), phalaropes (*Phalaropus* spp.), and sandpipers (*Calidris* spp.). Collared lemming and brown lemming also occur throughout the tundra.

### Spatiotemporal stratification

We used three landscape metrics for spatial stratification. First, as a measure of tundra productivity and the amount of vegetative cover versus bare rock, we used the Normalized Difference Vegetation Index (NDVI), which estimates the amount of photosynthetic activity occurring within a pixel based on the reflectance values of red and near infrared wavelengths [47]. NDVI is a good predictor of above ground estimates of vegetative productivity such as biomass, ecosystem respiration, and gross ecosystem productivity in various Arctic tundra ecosystems [48], [49]. We calculated NDVI using a 30 m resolution Landsat Thematic Mapper image taken on 18 July 2010 (United States Geological Survey http://earthexplorer.usgs.gov), which corresponds to the timing of vegetation green up and is consistent with the period used in a remote sensing study of shorebird breeding habitat in the same region [50]. Using a Geographic Information System (GIS; ArcMap 9.3.1, ESRI, Redlands, CA, USA) NDVI calculations were applied to pixels occurring on land only using land and water layers (Natural Resources Canada, http://www.geobase.ca). We then calculated the mean NDVI value within a 15×15 pixel roving window (450×450 m) and classified pixels with a binomial variable: either high (0.1–1) or low vegetative productivity (−0.13–0.1) (*N*: 0 =  low, 1 =  high). Pixels in the low productivity range were surrounded mostly by bare ground and exposed rock while those in the high range were surrounded by tundra vegetation (B.R. personal observation), which is consistent with studies using NDVI [51].

As a second landscape metric, we used a terrain ruggedness index derived from a 30 m resolution digital elevation map (Natural Resources Canada, http://www.geobase.ca). The index was calculated for each pixel as the standard deviation in elevation (m) of surrounding pixels in a 33×33 pixel roving window (990×990 m). Pixels were binomially classified as low (0–10 m) or high (>10–89 m) ruggedness (*R*: 0 =  low, 1 =  high).

The final landscape metric was the proportion of pixels classified as standing freshwater (i.e., excluding rivers or streams), which was based on the land and water GIS layers (30 m resolution) and a 33×33 pixel roving window. We binomially classified pixels as being surrounded by low (0–0.08) or high (>0.08–1.0) amounts of freshwater (*W*: 0 =  low, 1 =  high). The scale and break points for all variables were subjectively chosen so that the landscape was divided into ecologically distinct habitat types. We combined the above binomial metrics to create 8 strata from each unique combination of metric categories and applied this stratification to terrestrial areas (Table 2).

**Table 2:** Values of the landscape metrics for each of the 8 strata used to stratify the 2030^2^ study area located among the Coxe Islands, Igloolik Island and the northern tip of the Melville Peninsula, Nunavut, Canada.

Strata	Tundra productivity	Terrain ruggedness	Freshwater	Total area (km^2^)	Transects sampled		
	(*N*)	(*R*)	(*W*)		2010	2011	2012
1	low	high	low	148	12	18	12
2	high	high	low	24	0	17	12
3	low	high	high	79	9	14	11
4	high	high	high	11	0	6	8
5	low	low	high	76	17	16	12
6	low	low	low	182	14	11	10
7	high	low	high	98	1	27	19
8	high	low	low	123	2	11	7

See text for the high and low cut-off values used for each landscape metric.

Data on bird abundance was collected each summer from 2010–2012, allowing us to temporally stratify our data. Each summer we deployed a remote weather station (PortLog, Rainwise Inc., Bar Harbor, ME, USA) to collect daily mean temperatures and total rainfall. The summers of 2010 and 2011 were warmer and dryer than the mean for Igloolik from 1980–2000 (Figure 2). Conversely, summer 2012 had temperatures more similar to the 20-year mean, but received significantly more rainfall in June (Figure 2). To estimate spring snow cover throughout our study area, we used Moderate Resolution Imaging Spectroradiometer (MODIS) data (National Snow and Ice Data Center: http://www.nsidc.org), which classifies 250 m resolution pixels as snow or bare ground using the Normalized Difference Snow Index [52]. Using MODIS data from 18 June 2010, 17 June 2011, and 17–18 June 2012 (days with sufficiently cloud-free skies), we estimated the proportion of pixels within our study area classified as snow relative to bare ground; the majority of migratory birds breeding in our study area generally arrive by these dates [53]. Spring snow cover was 99% in 2010 and 2011, and 77% in 2012. We temporally stratified our abundance data based on daily mean temperatures, total summer rainfall, and spring snow cover, considering 2010 and 2011 to be warm and dry with high spring snow cover (*T* = 0), and 2012 to be cool and wet with moderate spring snow cover (*T* = 1).

We estimated annual lemming abundance for our study area with snap traps (Museum Special, Forestry Suppliers Inc., Jackson, MS, USA). Following a protocol similar to Gruyer et al. [54], we set snap traps along 4 transects greater than 100 m apart: 2 in a wet meadow habitat dominated by sedges (*Carex* spp.) and 2 in a dry mesic habitat dominated by *Dryas* spp. Each transect consisted of 20 stations 15 m apart with 3 snap traps within 2 m of each station (60 traps per transect). Traps were baited with peanut butter, set in the morning, and checked every 24 hours for 3 consecutive days (720 trap nights/year). Each morning all traps that had been triggered (either by misfire or catching an animal) were re-baited and re-set. Annual lemming abundance for the study area was expressed as the total number of lemmings caught along all transects per 100 trap nights; this metric of lemming abundance had a positive, linear relationship with lemming density in another study in Nunavut [21]. During 2011, we experienced a peak in lemming abundance (3.24 lemmings/100 trap nights), which was preceded by low lemming abundance in 2010 (0.29 lemmings/100 trap nights) and followed by a decline in 2012 (0.00 lemmings/100 trap nights). We also temporally stratified our bird abundance data by lemming abundance, considering 2010 and 2012 to be low (*L* = 0) and 2011 to be high (*L* = 1).

### Distance sampling design

To estimate the density of each guild, we used distance sampling to estimate a detection function, which predicts the probability of an individual being detected as a function of its perpendicular distance from the transect [55]. Each summer 30 new random start locations were generated >1 km apart within each stratum using GIS. We walked 1 km transects using a map, compass, and GPS unit to navigate from each start location, ensuring that we remained within a single stratum. Transects were straight lines when possible, but curved as needed to remain in a stratum and avoid water bodies [55]. Each year, we attempted to survey a minimum of ten transects per stratum from 12 July to 30 August (Table 2, Figure S1), which roughly encapsulated the time between hatching and southern migration for birds breeding in our study area [53]. No transect was surveyed more than once in a year. Transects were surveyed primarily on fair weather days with good visibility, low wind, and no precipitation. Each transect took between 15 and 45 minutes to survey depending on the number of birds observed. Surveys occurred from 06:00 to 21:00 and time of survey was tested as an explanatory variable in detection functions to account for potential variation in activity. During the breeding season, Arctic birds can be active for up to 12 hours per day and this activity can occur any time throughout the 24 hours of daylight [56]–[58], so timing of surveys is less critical than at more southern latitudes. Along each transect we recorded every bird observed along with its distance (laser range finder, Bushnell, Overland Park, KS, USA) and compass bearing relative to the observer's GPS location, which was used to estimate perpendicular distance to the transect using a GIS as required for distance sampling. All individuals were classified as songbird, shorebird, gull, loon, or goose and identified to species when possible (see Table S1 for a list of species observed). If multiple birds within the same guild occurred in a cluster at the same location, they were considered a single observation and cluster size was recorded.

Treating guilds separately and combining data across strata, we used Distance 6.0 release 2 software [59] and Akaike Information Criteria corrected for small sample size (AIC_c_) [60] to determine the most appropriate detection function and to parameterize the top models. We first estimated the detection functions with all observations and then truncated the data at the distance that predicted the probability of detection to be 0.15 [55]. We used the multiple covariate distance sampling engine in Distance, which allows for additional covariates in the detection function [61]. We tested whether the covariates time of day (0 =  morning/evening, 06:00–09:59 and 17:00–21:00; 1 =  midday, 10:00–16:59), date (before 7 August, 7 August – 18 August, after 18 August; represented with two dummy variables with after 18 August as the base category), or year (2010, 2011, or 2012; represented with two dummy variables with 2012 as the base category) the survey was conducted, or terrain ruggedness (0 =  low, 1 =  high) in which the transect was located, significantly improved the detection function fit. Thirty candidate models were compared for the detection functions (Table S2).

### Statistical analysis of spatiotemporal variation

Our goal was to determine whether our stratification variables significantly explained variation in the number of individuals observed within each guild (a measure of abundance). Because there is uncertainty and error associated with the density estimates produced by Distance, these density estimates cannot be used directly in statistical analyses. Therefore, we used a method for analysing designed experiments with distance sampling data, where treatment effects on abundances are of interest [62]. Using this approach, data from stratified distance sampling can be summarized as counts of animal clusters and mean cluster size within each stratum, along with each cluster's detection probability. Counts of clusters can then be modelled as a function of the strata variables in a generalized linear model with a Poisson error distribution. Using a log link function, variation in survey effort and detection probability across strata can be accounted for with an offset term in the Poisson model:
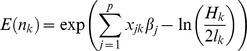
where *n_k_* is the count of clusters in strata *k*, *x_jk_* are the *p* different variables used to describe each strata *k*, *β_j_* are their associated coefficients, *H_k_* is the product of the mean cluster size for strata *k* and the total probability of a cluster being detected along a transect in strata *k* (estimated from the detection function), and *l_k_* is the total length of all transects surveyed in strata *k*; −ln(*H_k_*/2*l_k_*) is the offset [62].

Treating each guild separately, we used the original count data and AIC_c_ model selection to choose between competing *a priori* candidate models with the above form, each with different combinations of tundra productivity (*N*), terrain ruggedness (*R*), proportion of freshwater (*W*), weather (*T*), lemming abundance (*L*), and interactions as the *x_j_*'s. Because we surveyed only 3 transects in strata with high vegetation productivity (*N* = 1) in 2010 (Table 2), we did not include both *T* and *L* in any one candidate model for guilds we believed would be influenced by vegetation productivity (Table 3: shorebirds, songbirds and geese). By including only one of *T* or *L* in a model, data from 2010 was combined with data from either 2011 or 2012, respectively, which adequately increased the sample size of transects surveyed in strata with high vegetation productivity (Table 2). We used the ‘glm’ command in the statistical package R [63] to parameterize each candidate model. Because there is uncertainty in the estimation of the detection function, and hence the offset, the standard errors, confidence intervals and *P*-values for each parameter in the top models may be unreliable [62]. To account for uncertainty in the offset, we calculated bootstrap standard errors and 95% and 99% percentile confidence intervals for all parameters based on 999 nonparametric bootstrap resamples of transects within strata. We used Distance and R to analyse each bootstrap resample with the same method used for the original count data (see [62] for details). For each guild, we re-stratified transects using only the significant variables (based on bootstrap standard errors and the 95% percentile confidence intervals) in the top Poisson model. We then estimated bird density within these new strata using Distance with the appropriate detection function for each guild (Table 3).

**Table 3:** Detection function model forms determined to be most parsimonious (AIC_c_ a) for each avian guild.

	Truncation (m)						
Guild	Left	Right	Model form	Series expansion	# adjustment terms	Covariates	*P*-value^b^
Songbirds	0	50	hazard rate	n/a	0	Julian day	0.87
Shorebirds	0	40	half normal	cosine	1	n/a	0.57
Gulls	20	245	half normal	n/a	0	n/a	>0.99
Geese	0	375	half normal	n/a	0	year	0.90
Loons	50	260	half normal	n/a	0	n/a	0.72

a See Table S2 for details of the AIC_c_ analysis.

b *P-*values were obtained from a Kolmogorov–Smirnov test of the fit of the observation data to the detection function.

## Results

### Detection functions

For all guilds except songbirds, the half normal detection function model was chosen as the most parsimonious using AIC_c_; the hazard rate model was most parsimonious for songbirds (Table 3). Details of the AIC_c_ analysis and graphs of each detection function are presented in Table S2, Figure S2. The detection functions for each guild fit the data well (Table 3, Figure S2). The detection function for songbirds included date as a covariate, which predicted the probability of detecting a bird beyond 10 m from the transect was higher at the beginning of the post-hatching period and declined as the season progressed (Figure S2). The detection function for geese included year as a covariate, which predicted the probability of detection increased from 2010 to 2011 and then decreased in 2012 (Figure S2). Detection functions for the other guilds did not have any covariates (Table 3).

### Spatiotemporal variation

**Figure 3:**
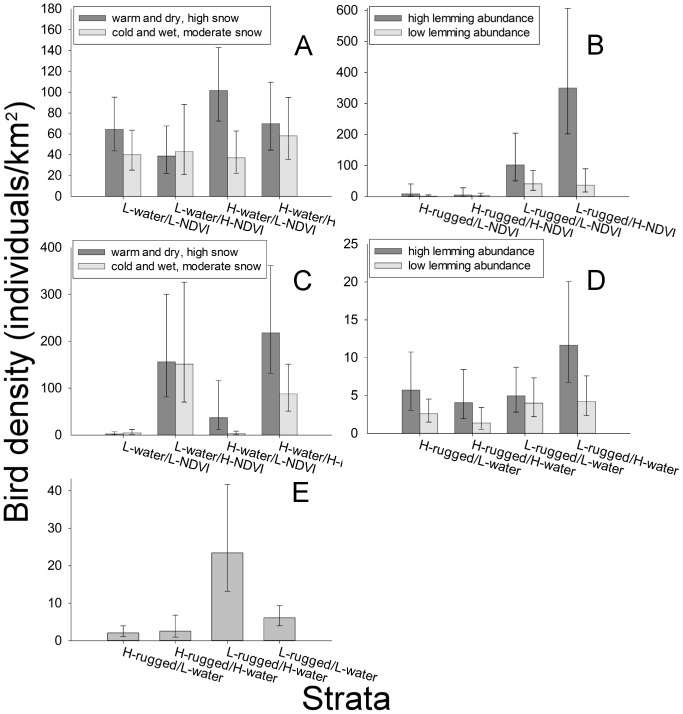
Guild-specific density estimates in relation to summer weather, lemming abundance, and landscape metrics. For each guild (A – songbirds, B – shorebirds, C – geese, D – gulls, E – loons) only variables found to significantly influence the number of individuals observed along transects were used (Table 4). The binomial landscape metrics (low (L) or high (H)) include the proportion of area made up of standing freshwater (water), terrain ruggedness (rugged) and vegetation productivity (NDVI). Note the scale on the density axis is different for each guild. Error bars show the 95% confidence interval around each density estimate.

For some guilds there was not overwhelming support for one top model, so we made inferences based on all models with ΔAIC_c_ values <2 (Table 4). There was strong evidence that songbirds were less abundant during the cool, wet summer with moderate spring snow cover (*T* = 1; see negative coefficients significantly different than 0 for the *T* term in Table 4; Figure 3A). Although the weather variable (*T*) was included in the top models for geese, the coefficient was not significantly different than 0. Abundance of shorebirds and gulls both increased significantly during the lemming peak (Table 4, Figure 3B and 3D).

**Table 4:** Top log-linear Poisson models predicting the counts of bird clusters observed along transects.

	ΔAIC_c_/Akaike weight			Coefficient±SE^a^	
Guild	Model 1	Model 2	Term	Model 1	Model 2
Songbirds	0.00/0.64	1.78/0.26	*T*	−0.80±0.22**	−0.62±0.29*
			*W*	0.39±0.17**	0.47±0.21**
			*N*	−0.45±0.22*	−0.46±0.21*
			*T×N*	0.65±0.35*	0.67±0.35*
			*T×W*	-	−0.31±0.33
			int.	−9.60±0.17**	−9.64±0.19**
Shorebirds	0.00/0.79	-	*L*	0.96±0.45*	-
			*R*	−3.00±0.80**	-
			*N*	−0.12±0.51	-
			*L×N*	1.31±0.70*	-
			int.	−10.18±0.35**	-
Loons	0.00/0.38	0.83/0.25	*L*	0.32±0.30	-
			*R*	−1.17±0.36**	−1.12±0.36**
			*W*	1.24±0.30**	1.27±0.30**
			*R×W*	−1.15±0.59*	−1.18±0.60*
			int.	−12.16±0.26**	−12.03±0.21**
Geese	0.00/0.72	-	*T*	0.52±0.56	-
			*W*	1.05±0.46*	-
			*N*	3.25±0.41**	-
			*T×W*	−1.59±0.75*	-
			cons.	−12.65±0.45**	-
Gulls	0.00/0.21	0.38/0.17	*L*	0.71±0.23**	0.78±0.24**
			*R*	−0.13±0.31	−0.57±0.24**
			*W*	0.43±0.28	-
			*N*	-	−0.30±0.23
			*R×W*	−0.87±0.49*	-
			int.	−12.69±0.27**	−12.33±0.24**

All terms used, except the intercept (int.), were categorical (0 =  low, 1 =  high) and include summer weather (*T*), lemming abundance (*L*), terrain ruggedness (*R*), amount of freshwater (*W*), and vegetation productivity (*N*). Only models with ΔAIC_c_ values <2 are shown.

a Bootstrap standard errors

*Coefficient estimate significantly different from 0 (α = 0.05) based on bootstrap percentile confidence intervals.

**Coefficient estimate significantly different from 0 (α = 0.01) based on bootstrap percentile confidence intervals.

Averaged across all summers, songbirds were more abundant in strata with high amounts of freshwater (*W = *1) and low vegetative productivity (*N* = 0) (Figure 3A). The significant *T×N* interaction term in both top models for songbirds, however, indicated that songbird abundance declined during the cool, wet summer with moderate snow cover (*T = *1), but only in strata with low vegetation productivity (*N* = 0) (Table 4, Figure 3A). Regardless of lemming abundance, shorebirds were more abundant in flat strata (*R = *0); shorebirds also had a positive association with vegetation productivity, but only during the peak lemming year (significant *L*×*N* interaction term; Table 4), which is consistent with the densities estimates produced in Distance (Figure 3B). Averaged across all summers, loons were more abundant in flat areas (*R = *0) with high amounts of freshwater (*W = *1). The significant *R×W* interaction term indicated that loon abundance increased with the amount of freshwater only in flat areas (Table 4, Figure 3E). Geese were more abundant in strata with high vegetative productivity (Table 4), which is consistent with density estimates (Figure 3C). For geese, the significant interaction between the weather variable and water (*T×W*) indicated they were more abundant in strata with high amounts of water during the warm, dry summers with high spring snow cover, but less abundant in these strata during the cool, wet summer with moderate spring snow cover (Table 4, Figure 3C). Finally, the significant *R×W* interaction term in the top model for gulls indicated that gulls were most abundant in flat areas with high amounts of water (Table 4, Figure 3D).

## Discussion

As predicted, the landscape metrics we considered explained significant variation in the abundance of multiple guilds of Arctic-breeding birds. By focusing only on landscape metrics that were important for each guild, we estimated guild-specific spatiotemporal variation in density. Our density estimates fell within the range of those reported in other Arctic studies [53], [64]–[66]. Breeding bird densities vary considerably across the Arctic, which has been attributed to variation in primary productivity correlated with mean annual temperature and latitude [67], [68]. Our study area in the Northern Arctic Ecozone had an intermediate density of birds with higher densities than the Arctic Cordillera to the north (e.g., Ellesmere Island, Nunavut; [67]) and lower densities than the sub-Arctic to the south (e.g., Cape Churchill, Manitoba; [69]).

The spatial variation in abundance we observed generally supported our hypotheses (Table 1) and matched what would be expected based on ecological knowledge of each guild. For example, loons that require large lakes for breeding and foraging [70] were most abundant in flat areas with high amounts of freshwater; gulls, which often breed on marshy hummocks, raised beaches, and inland tundra around large ponds [71], [72], were most abundant in the same habitats as loons. Shorebirds were most abundant in flat areas with high vegetative productivity, as predicted based on habitat preference [64]. The amount of freshwater present may not have influenced overall shorebird abundance because some species prefer well drained, sparsely vegetated tundra (e.g., American golden-plovers), while others prefer wet, marshy habitats (e.g., sandpipers and phalaropes) [73].

By explicitly controlling for spatial variations in bird abundance, we were also able to determine how annual changes in weather, spring snow cover, and lemming abundance created more complex spatiotemporal patterns. During the warm, dry summers with high spring snow cover, songbirds were most abundant in rocky habitats with low vegetative productivity and high amounts of freshwater, which matches the preferred nesting habitat of Arctic songbirds [67]. During the cool, wet summer songbird abundance declined, but this response was less pronounced in strata with high vegetation productivity, where foraging opportunities for insectivores and granivores is greatest [74]. In Alaska and Scandinavia, cold conditions during precipitation events decreased daily arthropod activity, reducing foraging opportunities for insectivorous birds and resulting in negative demographic consequences [25], [26]. Cold temperatures, high wind and precipitation can reduce arthropod activity and abundance across the Canadian Arctic [75]. The bulk of Arctic-breeding songbirds' diet consists of arthropods [75], [76], so declining arthropod availability associated with cool, wet weather may have driven the spatiotemporal variation in songbird abundance observed in our study.

Regardless of weather conditions and spring snow cover, herbivorous geese were most abundant in areas with high vegetative productivity, which likely afforded the best foraging opportunities. Overall, goose abundance was not significantly influenced by weather and snow cover, but there was a correlation between weather conditions and the spatial distribution of geese relative to the amount of freshwater. Geese redistributed themselves from dryer areas in the cool, wet summer to wetter areas in the warm, dry summers, which may have been an antipredator strategy; Lecomte et al. [77] found incubating geese that were required to travel far distances to access water had a higher chance of losing their eggs to predation than those breeding in close proximity to water. Predation pressure may explain why lower spring snow cover and high amounts of rainfall, which generally benefit Arctic-breeding geese [20], [31], [32], did not result in an increase in goose abundance. The cool, wet summer with moderate snow cover coincided with a sharp decline in lemming abundance. Arctic fox predation on goose eggs increases dramatically when lemmings decline after a peak year [38], [44], which may have offset any increase in nest success associated with favourable spring conditions.

Although goose eggs are preferred alternate prey for Arctic foxes, shorebird eggs are consumed incidentally making predation risk on shorebird nests highest where geese are most abundant, particularly when lemmings are scarce [42]. Shorebird abundance declined with lemming abundance throughout our study area, but this pattern was most pronounced in habitats with high vegetation productivity, where geese were most abundant. Incidental predation of shorebird eggs by Arctic fox may have been the mechanism that drove reductions in shorebird abundance during crashes in the lemming cycle observed in this and other studies [41], [43].

Gulls also fluctuated in concert with lemming abundance. Similar to shorebirds, the eggs of Sabine's gulls are preyed on by Arctic foxes [78], which may have contributed to the decline in gull abundance while lemmings were scarce. Glaucous gulls and, in particular, long-tailed jaegers (*Stercorarius longicaudus*) consume large numbers of lemmings during peaks years [79], [80], which may have resulted in a positive numerical response for these species [81].

This study demonstrated that low lemming abundance and cool, wet weather were correlated with declines in multiple avian guilds. Because both of these unfavourable conditions were present during the summer of 2012, songbirds and shorebirds likely experienced high predation risk and low forage availability as poor weather reduced arthropod activity and abundance. Although lemming abundance was also low in 2010, weather was warm and dry and predator abundance was likely lower than 2012, which followed a lemming peak. Predators exhibit a positive numeric response during lemming peaks [81], [82], so predation risk toward birds is highest during the lemming declines that follow, when predators are abundant and their primary prey are unavailable [38], [40], [44]. The amount of summer rainfall in the eastern Canadian Arctic has increased over the last 30 years [83] and the amplitude and frequency of lemming peaks is declining in other circumpolar regions [45]. Both of these patterns are predicted to intensify under various climate change scenarios [8], [45], which may have negative impacts on the productivity of songbirds, shorebird, gulls and possibly geese.

Given the results of this and other studies, predicting how climate change will impact the diversity and abundance of birds in the Arctic remains challenging [84]. Temperature increases will likely lead to longer growing seasons along with increases in primary production and arthropod abundance [21], [85], [86]. Warmer ecosystem may, therefore, support a higher density of shorebirds, songbirds, and geese, particularly as species disperse further north [87], [88]. However, increases in summer rainfall [8], which reduces foraging opportunities for insectivores, may negate advantages gained by warmer weather. Heavy summer rainfall can directly cause nestling mortality of Arctic-breeding raptors [83], [89], but the potential indirect effect of heavy rainfall on raptors through reductions in their avian prey should also be considered. Warmer temperatures and increased rainfall also advance spring snow melt, creating benefits for geese [20], but potentially leading to phenological mismatch between peaks in arthropod abundance and hatching of insectivores [17]. Shifts in winter weather and snow conditions associated with climate change are affecting the lemming cycle [90], which is clearly linked to the productivity of Arctic-breeding birds. Although the short-term nature of this study and lack of replication across multiple cool, wet summers limits our ability to make long-term predictions, we provide an example of a relatively simple way to monitor the correlation between weather, spring snow cover, lemming abundance, and spatiotemporal variations in a diversity of Arctic-breeding birds.

## Supporting Information

Figure S1 Frequency distribution of transects surveyed within 5-day periods by strata. Frequencies shown represent the total number of transects surveyed across years (2010–2012), because relative timing of surveys was consistent throughout the study.
(PDF)

Figure S2 Estimated detection functions (red lines) and frequency histograms of the actual number of birds observed at different distances from transects (blue bars). If a covariate was included in the detection function for a guild, a different detection function is shown for each value of the covariate. Transects were located on the Coxe Islands, Igloolik Island, and the northern tip of the Melville Peninsula, Nunavut, and surveyed from 12 July – 30 August, 2010–2012.
(PDF)

Dataset S1 Raw distance sampling data. Descriptions of fields and species codes are given in the Meta Data and Species Codes tabs, respectively.
(XLSX)

Table S1 List of avian species observed along transects. Transects were located on the Coxe Islands, Igloolik Island, and the northern tip of the Melville Peninsula, Nunavut, from 12 July – 30 August, 2010–2012.
(PDF)

Table S2 Details of the AICc analysis used to choose the most appropriate detection function for each avian guild. Birds were surveyed on the Coxe Islands, Igloolik Island and the northern tip of the Melville Peninsula, Nunavut, from 2010–2012. The ΔAICc value of the model used for each guild is bolded. If multiple models had a ΔAICc <4, the model with the least number of parameters was chosen to satisfy the rule of parsimony. Models with no ΔAICc value (-) did not converge during parameter estimation.
(PDF)
