# Supplementary material for: The Influence of Weather and Lemmings on Spatiotemporal Variation in the Abundance of Multiple Avian Guilds in the Arctic
Source: PLoS One. 2014 Jul 1;9(7):e101495. doi: 10.1371/journal.pone.0101495 (PMC4077800; doi:10.1371/journal.pone.0101495)
Supplement: Figure S1 — Frequency distribution of transects surveyed within 5-day periods by strata. Frequencies shown represent the total number of transects surveyed across years (2010–2012), because relative timing of surveys was consistent throughout the study. (PDF) [file pone.0101495.s001.pdf]

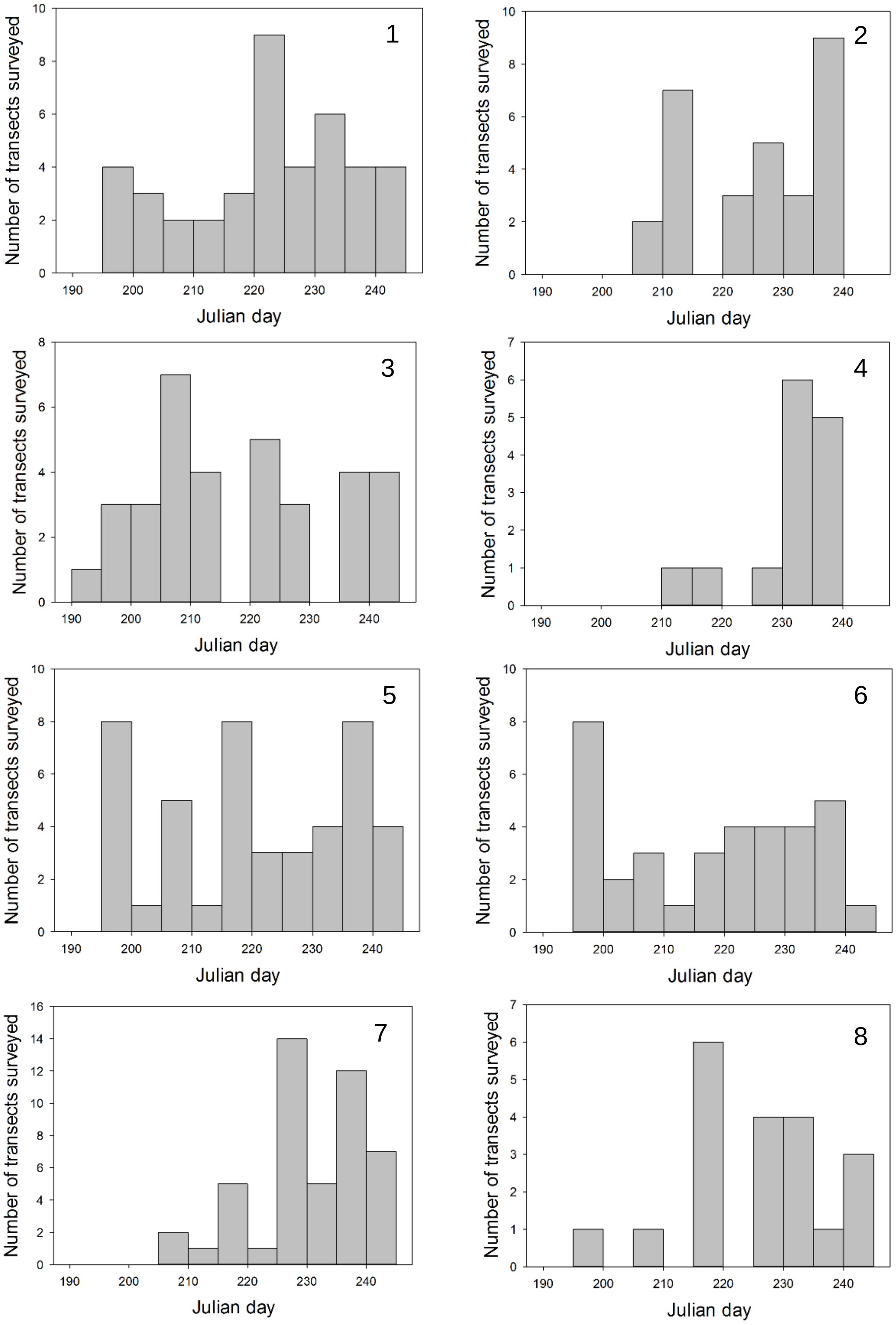


**Figure S1. Frequency distribution of transects surveyed within 5-day periods by strata.** Frequencies shown represent the total number of transects surveyed across years (2010-2012), because relative timing of surveys was consistent throughout the study.
