# Supplementary material for: The Influence of Weather and Lemmings on Spatiotemporal Variation in the Abundance of Multiple Avian Guilds in the Arctic
Source: PLoS One. 2014 Jul 1;9(7):e101495. doi: 10.1371/journal.pone.0101495 (PMC4077800; doi:10.1371/journal.pone.0101495)
Supplement: Figure S2 — Estimated detection functions (red lines) and frequency histograms of the actual number of birds observed at different distances from transects (blue bars). If a covariate was included in the detection function for a guild, a different detection function is shown for each value of the covariate. Transects were located on the Coxe Islands, Igloolik Island, and the northern tip of the Melville Peninsula, Nunavut, and surveyed from 12 July – 30 August, 2010–2012. (PDF) [file pone.0101495.s002.pdf]

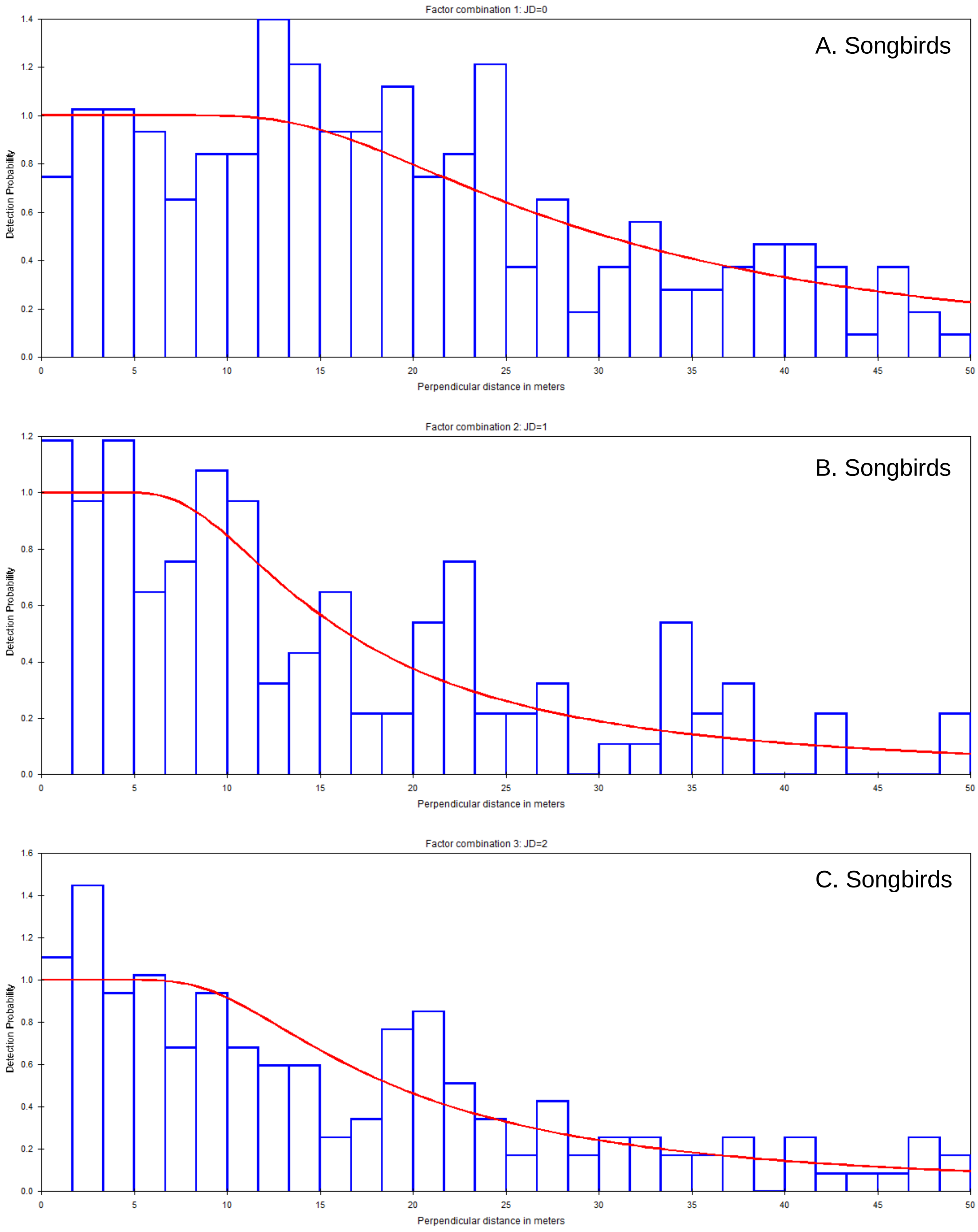

Figure S2. Estimated detection functions (red lines) and frequency histograms of the actual number of birds observed at different distances from transects (blue bars). If a covariate was included in the detection function for a guild, a different detection function is shown for each value of the covariate (value shown above the graph). Transects were located on the Coxe Islands, Igoolik Island, and the northern tip of the Melville Peninsula, Nunavut, and surveyed from 12 July – 30 August, 2010 – 2012.

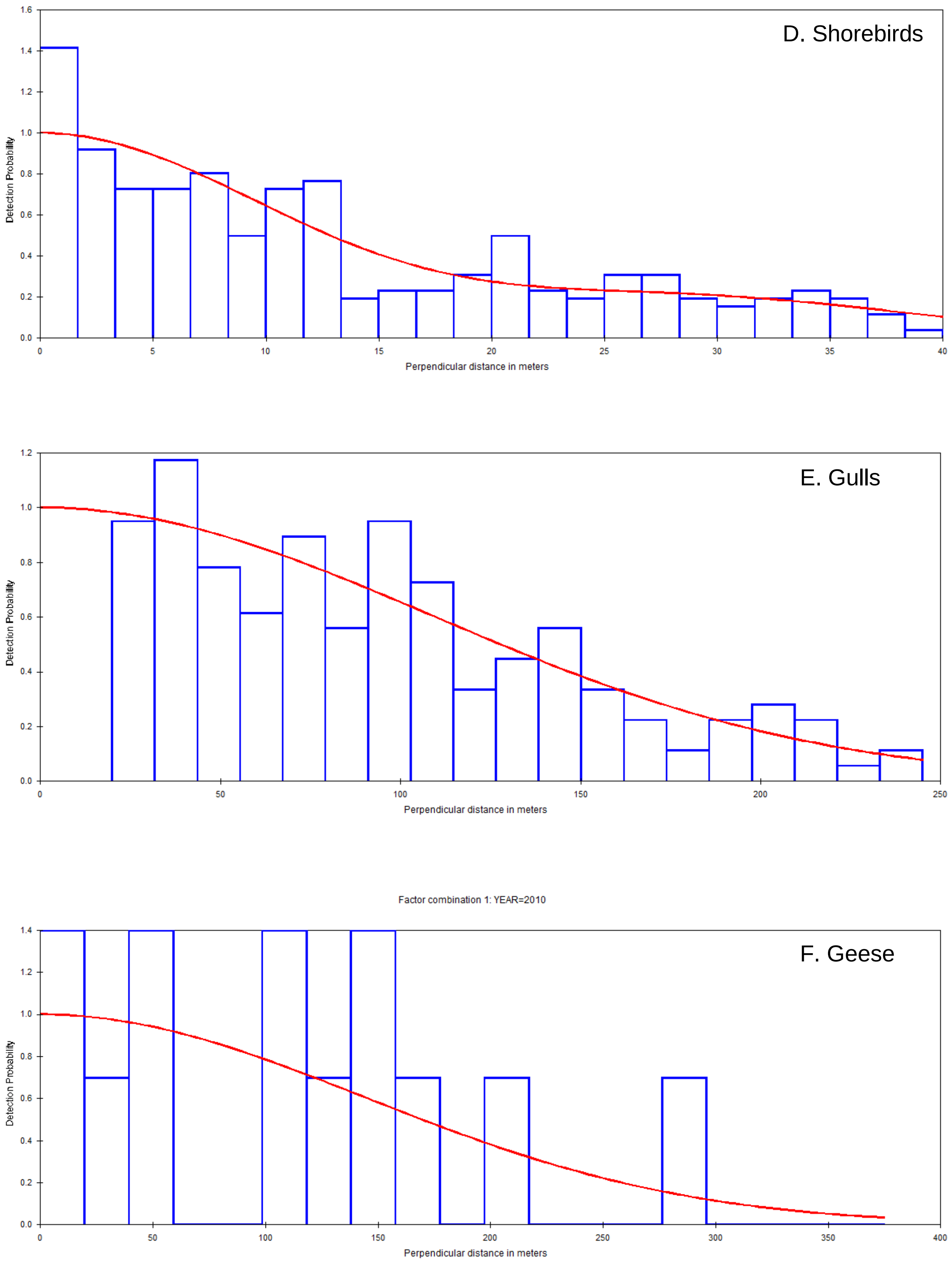

Figure S2. cont.

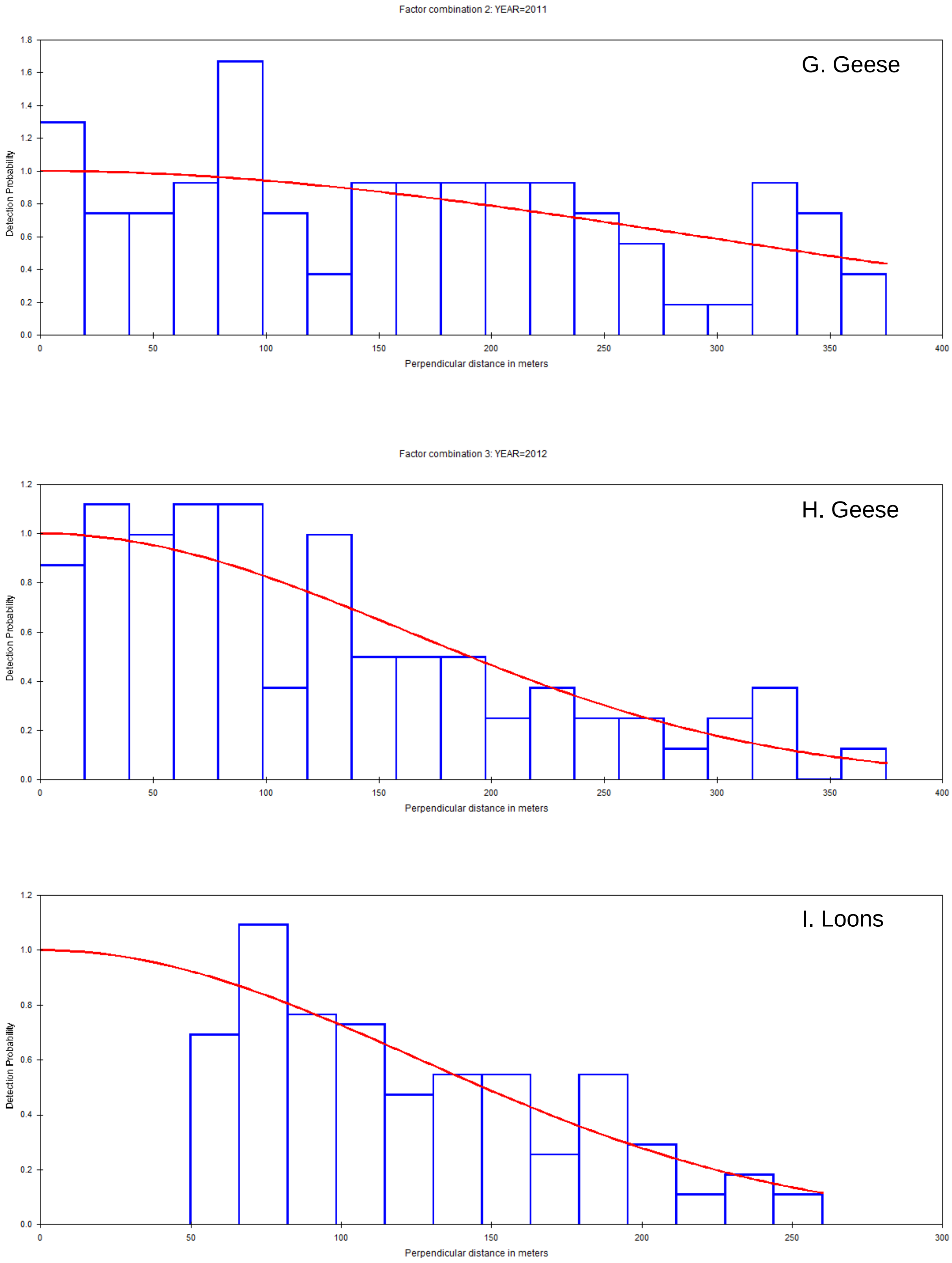

Figure S2. cont.
