# Supplementary material for: The Influence of Weather and Lemmings on Spatiotemporal Variation in the Abundance of Multiple Avian Guilds in the Arctic
Source: PLoS One. 2014 Jul 1;9(7):e101495. doi: 10.1371/journal.pone.0101495 (PMC4077800; doi:10.1371/journal.pone.0101495)
Supplement: Table S1 — List of avian species observed along transects. Transects were located on the Coxe Islands, Igloolik Island, and the northern tip of the Melville Peninsula, Nunavut, from 12 July – 30 August, 2010–2012. (PDF) [file pone.0101495.s004.pdf]

Table S1. List of avian species observed along transects. Transects were located on the Coxe Islands, Igoolik Island, and the northern tip of the Melville Peninsula, Nunavut, and surveyed from 12 July – 30 August, 2010 – 2012.

Avian Guild	Common	Scientific
Songbirds	snow bunting	*Plectrophenax nivalis*
	horned lark	*Eremophila alpestris*
	Lapland longspur	*Calcarius lapponicus*
	American pipet	*Anthus rubescens*
Shorebirds	dunlin	*Calidris alpina*
	red phalarope	*Phalaropus fulicarius*
	red-necked phalarope	*Phalaropus lobatus*
	American golden plover	*Pluvialis dominica*
	black-bellied plover	*Pluvialis squatarola*
	semipalmated plover	*Charadrius semipalmatus*
	Baird’s sandpiper	*Calidris bairdii*
	buff-breasted sandpiper	*Tryngites subruficollis*
	purple sandpiper	*Calidris maritima*
	stilt sandpiper	*Calidris himantopus*
	white-rumped sandpiper	*Calidris fuscicollis*
Geese	brant	*Branta bernicla*
	snow goose	*Chen caerulescens*
	Canada goose	*Branta canadensis*
	tundra swan	*Cygnus columbianus*
Gulls	glaucous gull	*Larus hyperboreus*
	herring gull	*Larus argentatus*
	Sabine’s gull	*Xema sabini*
	Thayer’s gull	*Larus thayeri*
	parasitic jaeger	*Stercorarius parasiticus*
	long-tailed jaeger	*Stercorarius longicaudus*
	Arctic tern	*Sterna paradisaea*
Loons	common loon	*Gavia immer*
	Pacific loon	*Gavia pacifica*
	red-throated loon	*Gavia stellata*
	yellow-billed loon	*Gavia adamsii*
Other	sandhill crane	*Grus canadensis*
	long-tailed duck	*Clangula hyemalis*
	common eider	*Somateria mollissima*
	king eider	*Somateria spectabilis*
	gyrfalcon	*Falco rusticolus*
	peregrine falcon	*Falco peregrinus*
	black guillemot	*Cepphus grylle*
	rough-legged hawk	*Buteo lagopus*
	snowy owl	*Bubo scandiacus*
	rock ptarmigan	*Lagopus muta*
	common raven	*Corvus corax*
