# Supplementary material for: The Influence of Weather and Lemmings on Spatiotemporal Variation in the Abundance of Multiple Avian Guilds in the Arctic
Source: PLoS One. 2014 Jul 1;9(7):e101495. doi: 10.1371/journal.pone.0101495 (PMC4077800; doi:10.1371/journal.pone.0101495)
Supplement: Table S2 — Details of the AICc analysis used to choose the most appropriate detection function for each avian guild. Birds were surveyed on the Coxe Islands, Igloolik Island and the northern tip of the Melville Peninsula, Nunavut, from 2010–2012. The ΔAICc value of the model used for each guild is bolded. If multiple models had a ΔAICc <4, the model with the least number of parameters was chosen to satisfy the rule of parsimony. Models with no ΔAICc value (-) did not converge during parameter estimation. (PDF) [file pone.0101495.s005.pdf]

Table S2. Details of the AIC_c analysis used to choose the most appropriate detection function for each avian guild. Birds were surveyed on the Coxe Islands, Igloolik Island and the northern tip of the Melville Peninsula, Nunavut, from 2010-2012. The ΔAIC_c value of the model used for each guild is bolded. If multiple models had a $\Delta AIC_c < 4$, the model with the least number of parameters was chosen to satisfy the rule of parsimony. Models with no ΔAIC_c value (-) did not converge during parameter estimation.

	Series	# Adjustment		#	ΔAIC_c				
Key function	expansion	terms	Covariates	Parameters	Songbirds	Shorebirds	Gulls	Geese	Loons
Half Normal	-	0	-	1	9.80	15.75	**2.30**	6.39	**0.94**
Half Normal	-	0	Time	2	6.94	17.31	4.22	8.21	0.00
Half Normal	-	0	Rugged	2	9.81	16.73	0.00	6.46	1.74
Half Normal	-	0	Year	3	10.93	16.00	2.49	**0.00**	1.25
Half Normal	-	0	Julian Day	3	5.90	12.41	5.89	**3.71**	2.47
Half Normal	Cosine	1	-	2	8.66	**1.91**	4.28	7.56	2.93
Half Normal	Cosine	1	Time	3	6.04	3.55	6.21	9.41	1.95
Half Normal	Cosine	1	Rugged	3	8.71	3.09	2.00	7.76	3.71
Half Normal	Cosine	1	Year	4	9.94	2.89	4.49	1.70	7.89
Half Normal	Cosine	1	Julian Day	4	5.29	0.00	7.88	5.22	4.43
Half Normal	Hermite Poly.	1	-	2	11.71	17.66	4.29	8.35	2.94
Half Normal	Hermite Poly.	1	Time	3	-	-	-	-	-
Half Normal	Hermite Poly.	1	Rugged	3	-	-	2.00	-	-
Half Normal	Hermite Poly.	1	Year	4	-	-	-	-	-
Half Normal	Hermite Poly.	1	Julian Day	4	-	-	-	-	-
Hazard Rate	-	0	-	2	9.72	2.54	4.91	7.02	2.67
Hazard Rate	-	0	Time	3	9.74	8.00	6.64	8.85	5.37
Hazard Rate	-	0	Rugged	3	13.89	7.90	3.56	7.42	5.37
Hazard Rate	-	0	Year	4	13.01	7.58	7.17	3.86	5.56
Hazard Rate	-	0	Julian Day	4	**0.00**	7.67	8.57	5.14	7.27
Hazard Rate	Cosine	1	-	3	8.58	3.43	5.79	8.89	4.45
Hazard Rate	Cosine	1	Time	4	8.98	13.34	8.69	10.71	7.32
Hazard Rate	Cosine	1	Rugged	4	10.01	13.31	5.11	8.87	7.32
Hazard Rate	Cosine	1	Year	5	11.94	13.47	10.67	5.54	7.89
Hazard Rate	Cosine	1	Julian Day	5	-	13.27	10.69	7.56	9.19
Hazard Rate	Simply Poly.	1	-	3	11.44	2.89	6.62	9.02	4.25
Hazard Rate	Simply Poly.	1	Time	4	10.01	7.93	-	10.83	5.70
Hazard Rate	Simply Poly.	1	Rugged	4	14.82	8.10	5.16	9.32	5.77
Hazard Rate	Simply Poly.	1	Year	5	12.40	7.79	8.61	5.52	7.13
Hazard Rate	Simply Poly.	1	Julian Day	5	0.97	8.96	9.90	7.09	5.05
